# Simultaneous multiplexed materials characterization using a high-precision hard X-ray micro-slit array

**DOI:** 10.1107/S1600577515005378

**Published:** 2015-04-22

**Authors:** Fan Zhang, Andrew J. Allen, Lyle E. Levine, Derrick C. Mancini, Jan Ilavsky

**Affiliations:** a Materials Measurement Laboratory, National Institute of Standards and Technology, 100 Bureau Drive, Stop 6520, Gaithersburg, MD 20899, USA; b Department of Physics, Illinois Institute of Technology, 3101 South Dearborn Street, Chicago, IL 60616, USA; c Advanced Photon Source, Argonne National Laboratory, 9700 South Cass Avenue, Argonne, IL 60439, USA

**Keywords:** measurement-in-parallel, simultaneous *in operando* characterization, heterogeneous structure, uncertainty reduction, multiplexed materials characterization

## Abstract

The needs both for increased experimental throughput and for *in operando* characterization of functional materials under increasingly realistic experimental conditions have emerged as major challenges across the whole of crystallography. A novel measurement scheme that allows multiplexed simultaneous measurements from multiple nearby sample volumes is presented. This new approach enables better measurement statistics or direct probing of heterogeneous structure, dynamics or elemental composition. To illustrate, the submicrometer precision that optical lithography provides has been exploited to create a multiplexed form of ultra-small-angle scattering based X-ray photon correlation spectroscopy (USAXS-XPCS) using micro-slit arrays fabricated by photolithography. Multiplexed USAXS-XPCS is applied to follow the equilibrium dynamics of a simple colloidal suspension. While the dependence of the relaxation time on momentum transfer, and its relationship with the diffusion constant and the static structure factor, follow previous findings, this measurements-in-parallel approach reduces the statistical uncertainties of this photon-starved technique to below those associated with the instrument resolution. More importantly, we note the potential of the multiplexed scheme to elucidate the response of different components of a heterogeneous sample under *identical* experimental conditions in *simultaneous* measurements. In the context of the X-ray synchrotron community, this scheme is, in principle, applicable to all in-line synchrotron techniques. Indeed, it has the potential to open a new paradigm for *in operando* characterization of heterogeneous functional materials, a situation that will be even further enhanced by the ongoing development of multi-bend achromat storage ring designs as the next evolution of large-scale X-ray synchrotron facilities around the world.

## Abstract 2

A novel measurement scheme that allows multiplexed simultaneous measurements from multiple nearby sample volumes is reported.

## 1. Introduction   

Across a wide range of current scattering-based materials research, indeed across the whole of crystallography, the need for increased experimental throughput and for *in operando* characterization of functional materials under increasingly realistic experimental conditions have emerged as major challenges. These challenges can become particularly acute if the characterization requires high spatial, momentum-transfer or time resolution, any of which can starve the measurement of useable intensity, whether this be for neutrons, electrons or X-rays. This is even true for the third-generation synchrotrons that produce X-ray beams of high brilliance and currently serve as the mainstream synchrotron sources worldwide (Bilderback *et al.*, 2005 ▸). In any case, the demand for access to these sources substantially exceeds their availability, necessitating innovative schemes for increasing the experimental throughput. We propose using microfabricated pinhole or slit arrays with submicrometer fabrication precision for producing multiple small X-ray probes, with individual pixels in a suitable area detector serving as independent detectors. This multiplexing approach can allow multiple experiments to be performed in parallel, with the additional advantage of simultaneously probing numerous nearby regions of a single heterogeneous sample. Such measurements can provide an effective imaging capability for heterogeneous samples that incorporate spatial gradients in either structure or local dynamics. We acknowledge that this approach relies on an ‘inline’ arrangement of the sample, optical beamline components and the detector. Therefore, not all measurement techniques would benefit. In this article, we use an implementation of this multiplexing approach for ultra-small-angle X-ray scattering (USAXS) based X-ray photon correlation spectroscopy (XPCS) measurements as an example to illustrate the application and potential of such an arrangement.

The technique of XPCS, which probes the dynamic properties of matter by analyzing the temporal correlations of the intensity fluctuations of scattered coherent photons, is essentially an extension of dynamic light scattering to the X-ray regime (Sutton *et al.*, 1991 ▸; Brauer *et al.*, 1995 ▸; Dierker *et al.*, 1995 ▸). Over the past two decades, XPCS has not only led to significant progress in our understanding of a wide variety of important dynamic phenomena but has also shown great potential for impacting many areas of statistical physics. Prominent examples include studies of concentrated colloidal suspensions (Lurio *et al.*, 2000 ▸; Orsi *et al.*, 2012 ▸; Zhang *et al.*, 2013 ▸), glassy dynamics (Robert *et al.*, 2006 ▸; Guo *et al.*, 2009 ▸), atomic diffusion (Leitner *et al.*, 2009 ▸; Ruta *et al.*, 2012 ▸) and dynamical heterogeneities in soft matter (Trappe *et al.*, 2007 ▸; Guo *et al.*, 2011 ▸). Despite these successes, XPCS is severely constrained by the lack of coherent flux from a third-generation synchrotron source. To make use of the coherent portion of the X-ray beam, a common practice is to utilize a small pinhole with dimensions similar to the transverse coherence lengths of the X-ray undulator beam. This small pinhole serves as a secondary coherence source. However, this approach is very inefficient since the pinhole blocks nearly the entire incident beam, placing a strict limitation on the maximum coherent flux. Clearly, the use of multiple such apertures can do much to improve the efficient use of X-rays. Additionally, X-ray scattering cross sections are generally much smaller than those for light scattering. As a result, one general yet critical aspect of an XPCS experiment is the coherent signal-to-noise ratio (SNR); this tends to define the minimum counting time required to determine the temporal correlations of interest. This issue is linked to another major challenge faced by XPCS, which is that beam damage often needs to be minimized for low-*Z* (soft matter) materials, whose intrinsic dynamic time scales would otherwise make them ideal systems to be investigated by XPCS.

To overcome these challenges, many efforts have been devoted to optimizing XPCS measurements using third-generation synchrotron sources. These efforts include careful considerations that aim to increase SNR, such as making use of the beam at X-ray energies with the highest X-ray brilliance, developing beamline optics that preserve both beam brilliance and coherence, and choosing detectors that are efficient in detecting X rays and are able to resolve speckles on a time scale commensurate with the sample dynamics (Roseker *et al.*, 2011 ▸; Westermeier *et al.*, 2013 ▸). Recently, a new scheme, referred to as X-ray speckle visibility spectroscopy, has been introduced. This method, by linking X-ray contrast to relaxation time, shows promise for improving the accessible dynamic timescale to include the millisecond regime (DeCaro *et al.*, 2013 ▸). It is also worth noting that, for XPCS measurements, common methods undertaken to reduce beam damage, such as using flow cells or moving samples, do not apply due to the sample motion interfering with the measured dynamics.

Here, we show how a multiplexing scheme can benefit USAXS-XPCS, an inline XPCS technique, by simultaneously improving the measurement statistics and reducing radiation exposure of the sample. Using XPCS measurements of the equilibrium dynamics of a colloidal suspension as an example, we prove that, not only does such an array allow some of the challenges faced by XPCS measurements to be overcome, but also that the multiplexed measurement configuration offers the additional potential to map out or image any spatial variations in the temporal correlations that exist within a sample at any one time.

This paper is arranged as follows. We first describe the design and fabrication of the slit arrays. We then introduce the sample system used in this pilot study. A brief discussion of the USAXS-XPCS technique and modifications in its implementation to enable the multiplexed XPCS measurements is provided, followed by the experimental results that illustrate the potential of this approach. We conclude by offering our thoughts concerning the prospect of multiplexed measurements using slit arrays for both XPCS and other scattering, diffraction or spectroscopic techniques, particularly those based at X-ray synchrotron facilities.

## 2. Fabrication of slits   

**Figure 1:**
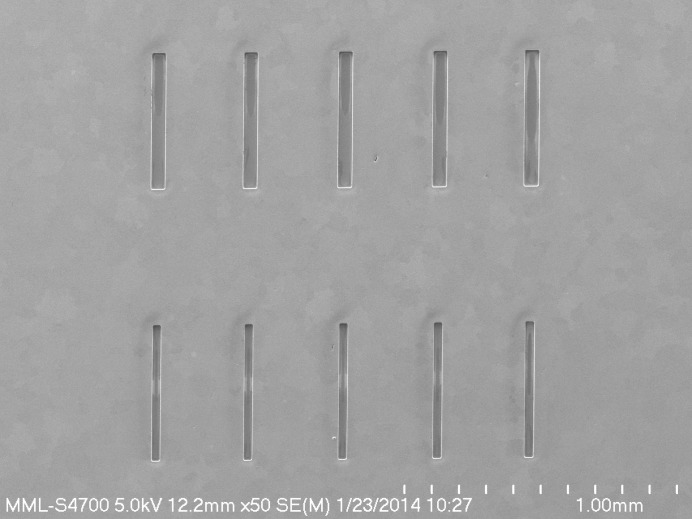
SEM image of slit arrays. For the multiplexed USAXS-XPCS measurements here, the multi-slit array comprised four of the five narrower (15 µm wide) bottom slits shown, with the USAXS entrance slit vertical aperture set to 50 µm to define the slit height.

Au slits suitable for use with hard X-rays were fabricated on 1 cm × 1 cm windows of 1 µm-thick low-stress Si_3_N_4_ held in a 500 µm-thick silicon frame. The windows were produced by optical lithography and etching of silicon wafers previously coated with Si_3_N_4_ using 30% KOH at 353 K (Seidel *et al.*, 1990 ▸). Very thin films (less than 20 nm each) of Cr followed by Au were evaporated onto the Si_3_N_4_ windows to provide a plating base with good adhesion. A 30 µm- to 40 µm-thick film of SU-8 negative photoresist was spin-coated onto the metal-coated Si_3_N_4_ windows and exposed through a mask of the slit pattern using a Karl Suss MA-6 UV optical mask aligner.^1^ After development, the patterned epoxy-based negative photoresist (EPON SU-8™) structures were used as a mold for Au electroforming. More than 25 µm of Au was plated onto the Si_3_N_4_ windows into the patterned SU-8 using electroplating solutions (Divan *et al.*, 2004 ▸). Au electroplating was performed in two stages using Techni-gold 25E (Technic Inc., Cranston, RI, USA) and Neutronex 309 (Enthone-OMI Inc., West Haven, CT, USA) baths, which operate at neutral and mildly acidic pH, respectively. The plating was carried out at a current density of 1 mA cm^−2^ with moderate agitation and continuous filtration of the solution. The first electroplating stage with the Techni-gold 25E solution produced a small-grain gold film for better adhesion to the substrate, but, as a result, this thin layer of gold (∼2 µm) was also under high tensile stress. We continued the plating in a second stage with the Neutronex 309 bath to obtain the final thickness of 25 µm to 30 µm. After electroplating, the SU-8 was stripped, leaving clear slit holes in the gold down to the Si_3_N_4_ windows. Electron micrographs (Fig. 1 ▸) of one such slit array show that the resulting slits are well formed with submicrometer precision, but with the minimal rounding of the corners typical of optical proximity lithography. The side-walls are straight, parallel and smooth with only nanoscale roughness. We note that it is critical to produce well formed slits due to the requirements to preserve X-ray coherence and minimize parasitic X-ray scattering. Fig. 1 ▸ also shows that some residual traces of resist appear to have remained on the bottom of the holes. This, however, is not expected to affect performance significantly, for reasons discussed later.

Through prior systematic variation of the aperture size, we established that a secondary coherent source size of 15 µm (horizontal) × 50 µm (vertical) provides the optimal signal-to-noise for XPCS measurements with the undulator A source at Sector 15 of the Advanced Photon Source (APS), Argonne National Laboratory. Consequently, we designed the slits specifically with this optimum slit size in mind. The slit spacing was chosen to ensure an avoidance of any cross-talk between the individual collimated incident beams defined by the slits. It was also important for the slits to match the size and periodicity of the sensors in a pixel array detector system so as to avoid any cross-talk between neighboring sensors in the detector array. For these experiments, the array included three rows of five slits. In each row, the slit spacing was 344 µm, the slit length was 500 µm, and the slit widths were 30 µm, 50 µm and 15 µm, respectively, in the three rows. Four of the 15 µm-wide slits were used for the results reported here with the USAXS entrance slit vertical aperture set to 50 µm to define the slit height.

## 3. Material system   

Spherical monodisperse silica powders were acquired from Bangs Lab, Fishers, IN, USA (catalog code: SS04N, lot #: 10750). The manufacturer-specified mean diameter of these microspheres was 1.01 µm, with a narrow size distribution having an associated standard deviation in diameter of less than 0.1 µm. A narrow size distribution of these microspheres is useful in understanding and interpreting the observed dynamics because the dynamic time scale of colloids strongly depends on the particle size. The density of the silica spheres is 2.0 g cm^−3^. The refractive index at a visible-light wavelength of 589 nm is between 1.43 and 1.46, which accounts for the milky appearance of the suspensions once prepared.

The stock silica powders were slightly aggregated. We used a mortar and pestle to gently break up the aggregates. The powders were then weighed and mixed with a mixed H_2_O and glycerol solvent, using deionized water and reagent-grade glycerol (ACS reagent, with 99.5% mass purity; Sigma-Aldrich, MO, USA), with an H_2_O:glycerol volume ratio of 1:3. The density of the solvent was 1.195 g cm^−3^. For the data presented in this article, the silica suspension had a silica volume concentration of 10%. To disperse the silica suspension, we repeated sonication and vortex mixing over a 48 h period. Previous results show that this preparation procedure produces a uniform dispersion of silica spheres with no detectable aggregation. We found that these silica suspensions remained well dispersed throughout the duration of the XPCS measurements.

## 4. Experiments   

**Figure 2:**
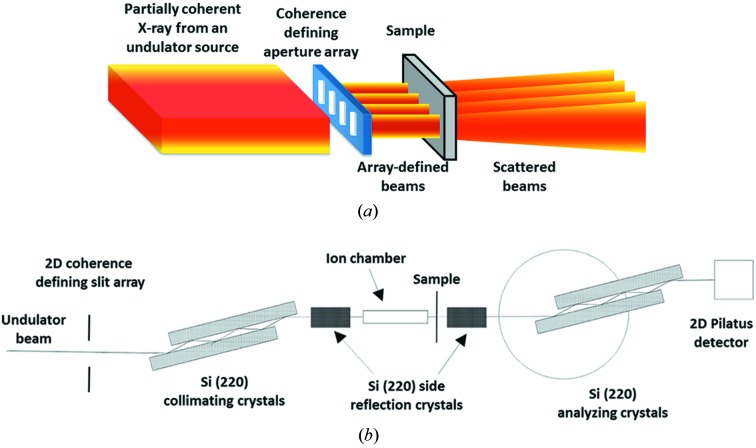
Instrumental configuration for slit-array-based multiplexed USAXS-XPCS measurements. (*a*) Schematic of the slit array serving as a set of secondary coherent sources. Each selects a different portion of the incident beam and consequently registers separate coherent scattering events. (*b*) Overall schematic of the multiplexed USAXS-XPCS instrument configuration.

We conducted the XPCS experiment using the USAXS instrument (Ilavsky *et al.*, 2009 ▸, 2013 ▸) at sector 15-ID of the APS. This mode of XPCS, referred to as USAXS-XPCS, takes advantage of Bonse–Hart crystal optics, and is capable of accessing a small *q* regime [where *q* = (4π/λ)sinθ, λ is the X-ray wavelength and θ is half of the scattering angle] that is inaccessible to conventional pinhole-camera-based XPCS instruments (Zhang *et al.*, 2011 ▸). USAXS-XPCS can be operated in two modes, a point-detection mode and a scan mode (Zhang *et al.*, 2011 ▸; Zhang, Allen, Levine, Ilavsky *et al.*, 2012 ▸). Similar to conventional XPCS, USAXS-XPCS in its point-detection mode has proven useful in studying equilibrium dynamics and revealing the *q*-dependence of the relaxation time scales (Zhang *et al.*, 2013 ▸). USAXS-XPCS in the scan mode can serve as a structural probe to study the time scales associated with non-equilibrium dynamics in materials under various conditions pertinent to material performance (Zhang, Allen, Levine, Espinal *et al.*, 2012 ▸; Zhang *et al.*, 2014 ▸). For the current multiplexing test study, we elected to examine a simple colloidal suspension using USAXS-XPCS in its point-detection mode. To incorporate the slit array, we modified the standard configuration for XPCS measurements, which was described previously (Zhang *et al.*, 2011 ▸). In particular, we positioned the slit array vertically in place of the coherence defining slits. The width of each multiplexed X-ray beam was set by the 15 µm width of the individual slits in the array. The vertical dimensions were adjusted using a set of highly polished horizontal JJ slits (JJ X-ray A/S, Lyngby, Denmark; model: IB-C22-HV vacuum X-ray slits) that comprise the standard incident beam slits for the USAXS instrument. For this experiment, we set the vertical slit size at approximately 50 µm, reflecting the anisotropic coherence of the incident X-ray beam from the undulator. Here, we used the bottom-edge of the JJ slits to block the residual traces of resist at the bottom end of the slit holes so that these traces did not affect the performance of the slits in the slit array. Additionally, it is known that the Bonse–Hart crystal optics are effective in removing parasitic scattering introduced by slits and other beamline optical elements positioned before the crystals (Xiao *et al.*, 2006 ▸). This property of the double-crystal optics, that is intrinsic to the USAXS configuration, further suppresses any scattering due to the imperfections of the slits. The instrument, as well as the implementation of the slit array as a set of secondary coherent sources, is illustrated in Fig. 2 ▸.

To take full advantage of the multiple coherent sources provided by the slit array, we also switched from a point detector (photodiode detector or scintillating detector) to a Dectris Pilatus detection system (Model: 100K-S, Dectris, Baden, Switzerland) (Eikenberry *et al.*, 2003 ▸) with 20 bit dynamic range and a pixel size of 172 µm × 172 µm. We aligned the relative positions of the slit array and the Pilatus detector so that at *q* = 0 the direct beams, after being transmitted through the slit array and the sample, were positioned on four separate pixels near the center of the Pilatus detector.

The colloidal-suspension sample was loaded into a standard liquid cell with a sample thickness of ∼1 mm. The XPCS measurements were conducted at room temperature and the X-ray energy was 10.5 keV (λ = 1.184 Å). Due to the equilibrium nature of the dynamics, we measured the intensity fluctuations at *q* values of 0.00015 Å^−1^, 0.0002 Å^−1^, 0.0003 Å^−1^, 0.0004 Å^−1^, 0.0005 Å^−1^, 0.0006 Å^−1^, 0.0007 Å^−1^, 0.0008 Å^−1^, 0.0009 Å^−1^ and 0.001 Å^−1^. For each *q*, we acquired a total of 2000 frames with a frame rate of 20 Hz, *i.e.* the measurement time resolution was 50 ms, and the measurement time at each *q* was ∼100 s.

## 5. Results and discussion   

**Figure 3:**
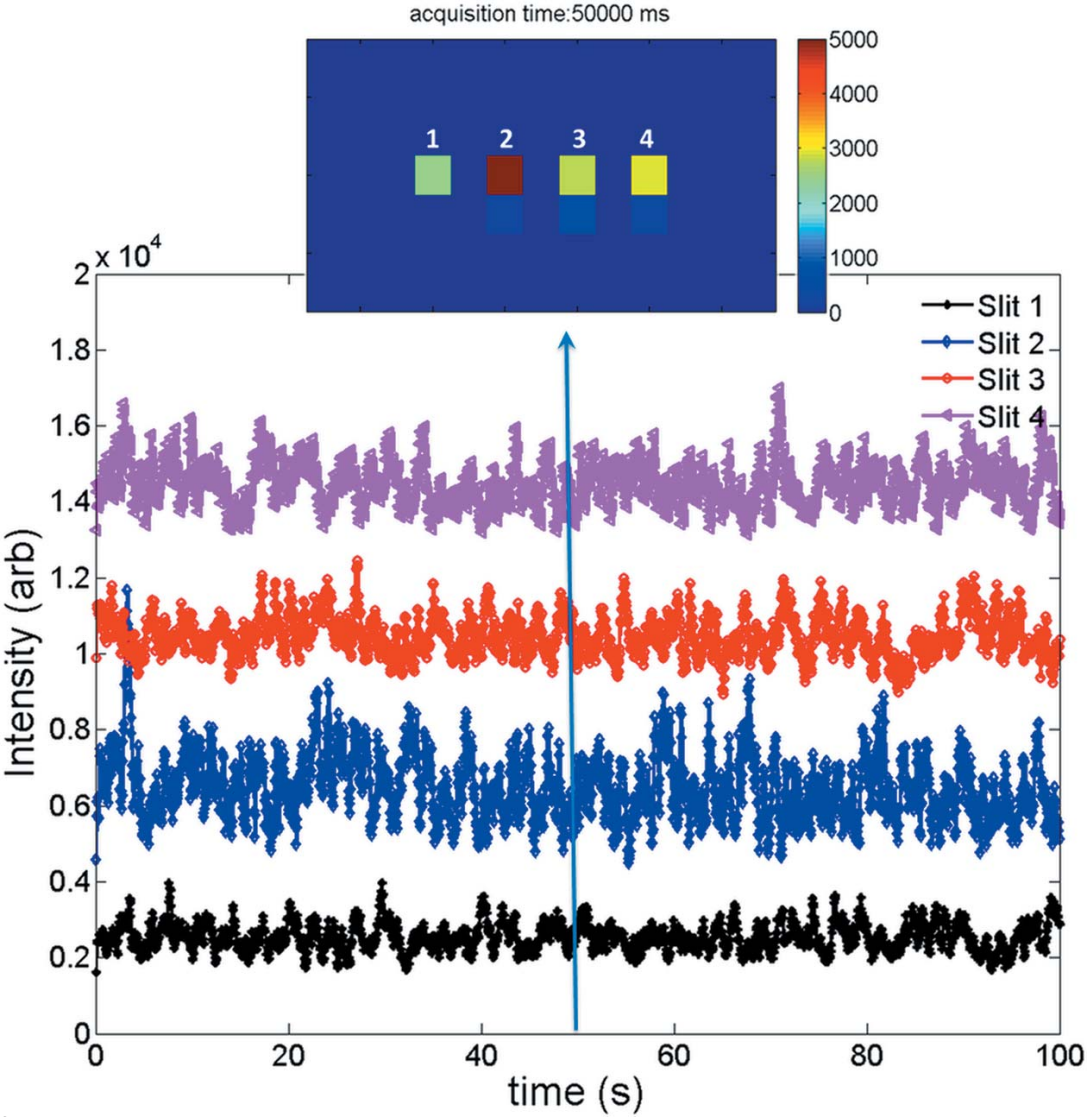
Scattering intensities as a function of time for 10% vol. silica spheres suspended in a 1:3 water:glycerol mixture at *q* = 0.0002 Å^−1^. Four sets of data correspond to scattering intensities acquired by four highlighted strips shown in the top panel, which were simultaneously acquired on the Pilatus detector at 50 s after the start of the measurement. The scattering intensities are displayed with offsets for viewing purposes. The time-dependent scattering data contain both the temporal fluctuations of interest and statistical noise due to uncertainties in the individual data intensity measurements. Provided the latter do not give rise to temporal correlations on the same time scale as those of interest, the correlations of interest can be separated using algorithms that incorporate the equations in the text.

We have developed a set of *MATLAB*-based programs to extract the individual pixel data from the Pilatus detector and analyze the observed time-dependent intensity fluctuations. The readout data, in principle, are simply two-dimensional intensity arrays. An example of such an array, acquired at 50 s after the start of the measurement with *q* = 0.0002 Å^−1^, is shown in the top panel of Fig. 3 ▸. In this case, the scattering intensities were mostly captured in the four highlighted pixels. We note that this was not always the case because, for this pilot study, we fixed the Pilatus detector on a detector stand. The slight change in the scattering angle, due to change in *q*, shifts the centroids of the coherent scattering intensity along the vertical direction. As a result, at some values of *q*, the centroids were captured by two vertically adjacent pixels. This spillover is illustrated by the faint signal directly below the pixels with highest intensity readouts. For the purpose of consistency, in our analyses, we integrated the coherent scattering intensities along a one-pixel-wide vertical stripe and assigned the integrated values as final scattering intensities. We also point out that, with modification to the instrumental controls, it is possible to synchronize the vertical position of the detector with the value of *q* so that the centroids are captured in single fixed pixels. This top panel also clearly shows that the horizontal spillover is minimal; we found that it was consistently less than 5% of the vertically integrated intensities, and therefore was neglected in our analyses.

We extracted the vertically integrated intensities for all of the acquired images, and examined the time-dependent intensity fluctuations corresponding to each slit in the slit array. An example of the intensity fluctuations for multiple slits on the slit array is shown in Fig. 3 ▸. Here, *q* = 0.0002 Å^−1^ and the intensities are offset for clarity. Dynamical intensity fluctuations are clearly visible in these data. Fig. 3 ▸ also suggests that the temporal behavior of these fluctuations was approximately constant, which is a characteristic of equilibrium dynamics. The four curves are not correlated with each other, which demonstrates that the four measurements were independent. Additionally, the magnitudes of the intensity ranges of these four curves were slightly different, which can be attributed to the different finish of the slits during fabrication and hence different coherent contrast. The absolute intensities in the four pixels were also different from each other, depending on how well each pixel was illuminated by the X-ray beam, but this was not correlated with the temporal fluctuations.

A proper analysis of XPCS intensity fluctuations originating from an equilibrium dynamic process requires calculation of the intensity autocorrelation function, which subsequently yields the characteristic relaxation times of the underlying process. Specifically, the intensity autocorrelation function, *g*_2_(*t*), is defined by (Grübel & Zontone, 2004 ▸; Dierker *et al.*, 1995 ▸)
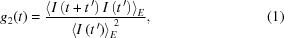
where *I*(*t*) is the integrated scattering intensity in an interval around a time *t*, and the angular brackets in (1) denote an ensemble average. This autocorrelation function is related to the intermediate scattering function of the sample, following
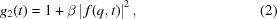
where *f*(*q*, *t*) = *S*(*q*, *t*)/*S*(*q*) is the normalized intermediate scattering function. Here, *S*(*q*) = *S*(*q*, 0) denotes the static structure factor and *S*(*q*, *t*) is the dynamic structure factor at time *t*. β is the optical contrast which, under ideal experimental conditions (*e.g.* fully coherent radiation and no readout noise), would be equal to one. In XPCS experiments, β takes a smaller value due to incoherent averaging introduced by the partially coherent X-ray beam, the geometrical configuration of the beamline, and readout noise. In our analysis, *I*(*t*) was the vertically integrated intensity, which was not normalized by the incident flux. We have previously shown that this normalization does not affect the measured dynamics (Zhang *et al.*, 2011 ▸).

The relaxation time represented by the calculated intensity autocorrelation function was further analyzed by modeling the autocorrelation function with a stretched exponential form [also known as the Kohlrausch–Williams–Watts (KWW) function (Caronna *et al.*, 2008 ▸; Madsen *et al.*, 2010 ▸; Zhang *et al.*, 2013 ▸)]. It is defined as
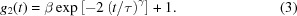
Here, τ is the characteristic relaxation time and γ is an exponent (the Kohlrausch exponent) which characterizes the shape of the autocorrelation function. The characteristic relaxation time τ is also the inverse of the relaxation rate, Γ. We note that the KWW function has been successfully employed in a wide range of dynamical investigations of soft material systems, such as colloidal dispersions and gels (Pontoni *et al.*, 2003 ▸; Duri *et al.*, 2009 ▸; Bandyopadhyay *et al.*, 2004 ▸; Bellour *et al.*, 2003 ▸; Cipelletti & Ramos, 2005 ▸; Fluerasu *et al.*, 2007 ▸), and is particularly useful for systems in which the particle diffusion does not follow simple Einstein–Stokes equations.

**Figure 4:**
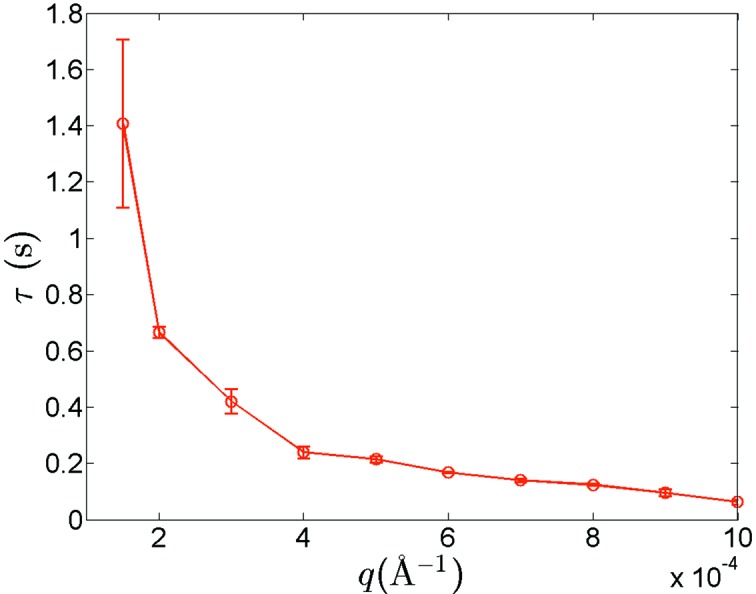
Relaxation time τ as a function of *q*, obtained from fits of the intensity autocorrelation function using equation (3). Vertical bars represent the computed standard deviation uncertainties for the relaxation time determined at each *q* among the multiplexed results acquired using four individual slits.

We analyzed the relaxation times for the silica suspension at different values of *q*, based on equation (3). For each *q*, our analyses yielded four relaxation times, one associated with each detector pixel. We further performed a simple statistical analysis of these relaxation times, and found the *q*-dependent statistical mean and standard deviation of the four relaxation times. This dependence is shown in Fig. 4 ▸. Here, we found that the relaxation time τ shows a monotonic decay as *q* increases, which shows that faster dynamics are associated with larger scattering vector *q*, an expected phenomenon considering that the short-range local fluctuations (large *q*) occur more rapidly than long-range fluctuations (small *q*) in interacting colloidal suspensions. We note that this set of results is very similar to previous findings for a suspension of polystyrene spheres of the same size and same volume concentration in glycerol, with the notable difference being that the degree of uncertainty is greatly reduced in the current study (Zhang *et al.*, 2013 ▸). This improvement in the measurement quality is a clear benefit provided by the multiple slit arrangement.

**Figure 5:**
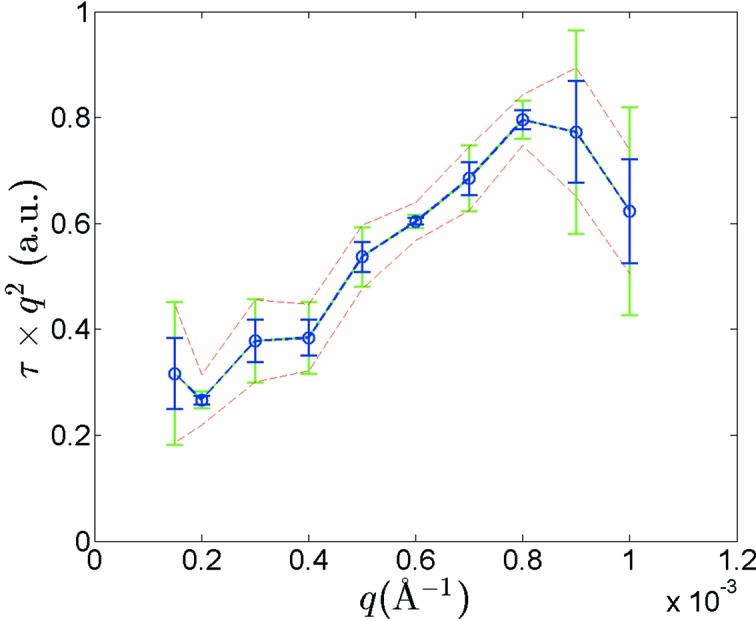
Dependence of the inverse of the effective diffusion coefficient on *q*. Green and blue vertical bars at each point represent the standard deviation uncertainties associated with a single measurement and four multiplexed measurements, respectively. The upper and lower bounds, defined by the dashed lines, show the uncertainties introduced by the finite *q* resolution of USAXS-XPCS.

We also inspected the inverse of the effective diffusion coefficient, which is proportional to τ*q* ^2^. Its dependence on *q* is displayed in Fig. 5 ▸, where we found that the inverse of the effective diffusion coefficient exhibits a peak that mimics a static structure factor. Similar to previous findings for polystyrene microspheres in glycerol, this result suggests de Gennes narrowing at interparticle length scales (Segrè & Pusey, 1996 ▸), and reflects the fact that strong fluctuations for values of *q* near the peak in *S*(*q*) take longer to decay, and therefore that the lowest free-energy configuration in the static case has a long life-time (Philipse & Vrij, 1988 ▸; Phalakornkul *et al.*, 1996 ▸; Falus *et al.*, 2006 ▸).

From a metrology point of view, we calculated three different sets of standard deviation uncertainties, as shown in Fig. 5 ▸. The first set (blue bars), calculated as σ(τ)*q* ^2^, where σ(τ) is the standard deviation uncertainty displayed in Fig. 4 ▸, shows how the statistical uncertainty due to the multiplexing arrangement translates to τ*q* ^2^. The second set (green bars) shows the statistical uncertainty associated with a single measurement. The dashed lines, on the other hand, were calculated following Δ(τ*q* ^2^) = σ(τ)*q* ^2^ + 2τ*q*Δ*q*, with the finite *q* resolution (Δ*q*) taken into account. It is apparent from Fig. 5 ▸ that, other than in the high *q* region where the quadratic term in Δ(τ*q* ^2^) dominates, the statistical uncertainty in the average of the four multiplexed-XPCS results is well bounded by the systematic measurement uncertainty. For most *q* values measured, the standard deviation of a single measurement is greater than or very close to the uncertainty associated with the finite *q* resolution. Thus, for the overall measurement times used here, despite only using four slits, the average multiplexed result provides the *q*-dependence within the uncertainties of the finite *q*-resolution available, whereas a single-slit measurement would not. This point illustrates how a multiplexed USAS-XPCS configuration can improve the quality of USAXS-XPCS measurements.

## 6. Conclusions and outlook   

In this paper, we have proposed a new experimental scheme that would enable multiplexed synchrotron X-ray measurements. This scheme relies upon the nanoscale precision that optical lithography provides, and takes advantage of the high flux density that a third-generation synchrotron offers. We have demonstrated the applicability of this scheme *via* multiplexed USAXS-XPCS measurements of a concentrated colloidal suspension. In this pilot study, each engineered-pinhole–detector-pixel pair has been used to conduct an independent point-detector temporal intensity-correlation measurement. For a sample where no spatial variation of the dynamics is expected, this would produce four independent measurements (in this case) of the mean relaxation time (for example). At the very least, this will provide a more reliable measurement of the temporal characteristics of the sample. Alternatively, if spatial variation of the temporal characteristics is present, it will be possible to map out or image aspects of the temporal variations (*e.g.* the mean relaxation time) with sample position. Using the colloidal suspension above as an example, the 1σ uncertainty in the relaxation time τ (0.21 s) was ∼0.01 s at *q* = 0.0005 Å^−1^. Thus, if XPCS measurements at two different positions show a difference of dynamic time scale of 0.03 s or above, there is a 99.7% or more chance that the measured dynamics are spatially heterogeneous. This would have major advantages for a sample with a gradient or other non-uniform microstructure, or in the case where some spatially heterogeneous dynamic state existed across the sample. We note that these advantages would also extend to the USAXS-XPCS scanning experiments of non-equilibrium dynamics where each engineered-pinhole–detector-pixel pair would be used to conduct an independent USAXS-XPCS scanning measurement. For the future, the global synchrotron community is embracing and developing the so-called multi-bend achromat (MBA) storage ring design, which promises to increase the X-ray beam brightness by two to three orders of magnitude beyond the current capability of major third-generation synchrotron sources. The coherent flux will be increased by 100 to 1000 times. With these improvements in both beam brightness and coherence, microsecond time resolution becomes possible for USAXS-XPCS. This drastic improvement in both X-ray coherence and flux will also enable XPCS experiments at higher X-ray energies, where the penetration power of X-rays allows *in operando* characterization of complex dynamics in realistic sample environments. Making use of the multiplexing scheme, this will enable unprecedented simultaneous characterization of heterogeneous non-equilibrium dynamics in a wide range of materials.

We note that, in principle, this multiplexing scheme can be applied to all in-line collimated techniques to enable simultaneous selected-area characterizations of heterogeneous structures or microstructures. In the context of the X-ray synchrotron research community, such techniques include ultra-small-angle X-ray scattering, analyzer-based X-ray diffraction, X-ray reflectivity, X-ray absorption spectroscopy (XAS) with reduced sample-to-detector distance, among others. For example, use of a slightly larger aperture size, paired with small spatially separated clusters of pixels on the two-dimensional detector would allow multiple USAXS measurements at several sample positions (or separate small samples) to be made at the same time. Each dataset would be collected simultaneously at the same successive *q* value for all of the sample apertures, permitting a dramatic enhancement of the measurement efficiency and accuracy. We also note that two-dimensional arrays of apertures can be used in place of the slit array used in this pilot study. In the case of an MBA lattice, these two-dimensional arrays can also serve as metrological calibration tools to systematically quantify the spatial properties of the coherent X-ray beams.

While utilization of slit and aperture arrays effectively reduce the overall X-ray photon flux that a sample receives and may lead to an increase in the measurement time for photon-starved techniques, it does offer the possibility of probing structural, dynamical or elemental inhomogeneities frequently found in a wide range of important functional materials, including catalysts, solid oxide fuel cells, membrane-based electrode materials and artificial bones. Furthermore, this scheme creates the opportunity to monitor the evolution of structures, dynamics and elemental compositions of the aforementioned inhomogeneities *in situ* and *in operando*. Different components of the same sample may not have the same response to an external stimulus, such as temperature, pressure and stress. To correctly and fully understand the response of different components of a heterogeneous sample under *identical* experimental conditions, it is critical that the measurements are conducted *simultaneously*. One example is the *in situ* characterization of dendrite formation in Li ion batteries, where understanding of the localized and heterogeneous formation of dendrites is of critical importance for the performance and safety of the batteries (Goodenough & Kim, 2009 ▸).

We note that, with modifications, this multiplexing scheme should be applicable to other forms of scattering-based research and crystallography. For example, it could be extended to neutron crystallography for the simultaneous characterization of suitable heterogeneous structures, where heterogeneity is found over millimeter length scales. We also note the current development of so-called VSANS instruments at neutron facilities around the world where multiple detector systems are employed for simultaneous measurements, that may then be separated out using multi-channel deconvolution algorithms. (Dewhurst, 2014 ▸; Andersen & Barker, 2014 ▸). It is within the context of these and related measurement-in-parallel approaches that we believe that the multiplexing scheme proposed in this article has the potential to open a new paradigm for *in operando* characterization of heterogeneous functional materials.

## Footnotes

1 Certain trade names and company products are mentioned in the text or identified in illustrations in order to specify adequately the experimental procedure and equipment used. In no case does such identification imply recommendation or endorsement by National Institute of Standards and Technology, nor does it imply that the products are necessarily the best available for the purpose.
